# Comparison of CHO cell cultivation and mAbproduction in the Allegro™ XRS 20 and conventional rocker type single-use bioreactors

**DOI:** 10.1186/1753-6561-9-S9-P59

**Published:** 2015-12-14

**Authors:** Gail Henry, Byron Rees, Charles Golightly

**Affiliations:** 1 Pall Life Sciences, 5 Harbourgate Business Park, Southampton Rd, Portsmouth, UK, PO6 4BQ

## Background

The Allegro XRS 20 bioreactor system is a single-use bioreactor system suitable for applications ranging from general life sciences research to seed train operations as well as small scale production at the 2 to 25literscale. It features a 3D rectangular biocontainer that is rocked across two independent axes perpendicular to one another.

These features allow for significant improvements in mixing and kLa when compared with a conventional rocking bioreactor that features a 2D "pillow" biocontainer that rocks across a single axis. The Allegro XRS 20 controller is able to control agitation of the biocontainer over a wide range of conditions, from gentle agitation required for shear sensitive cultures, to more vigorous conditions generating higher oxygen transfer rates and decreased mixing times needed for the high productivity systems seen in the biopharmaceutical industry today. Control of pH and DO is fully automated by the optical sensors supplied with the biocontainer. Gassing strategy is managed by three independent mass flow controllers and fluid additions can be made via three integrated pumps. All operations are managed through a user friendly touch screen interface.

## Materials and methods

Table 1 summarises set points and operating parameters to cultivate CHO cells producing monoclonal antibody in the Allegro XRS 20 bioreactor and a conventional rocker type bioreactor.

**Table 1:** Summary of Allegro XRS 20 Bioreactor operating conditions.

	XRS 20 Bioreactor	Conventional Rocking Bioreactor
Start Agitation (5 L)		
• Rate (CPM)	25	25
• X Angle (Deg)	5	N/A
• Y Angle (Deg)	5	8

Agitation (14 - 20 L)		
• Rate (CPM)	35	42
• X Angle (Deg)	5	N/A
• Y Angle (Deg)	15	10

Aeration Rate (L/min)	1.0	1.0

Seed Density (10^6 ^Cells/mL)	0.3 ± 0.1	0.3 ± 0.1

pH Set Point	7.2	7.2

Do Set Point (%)	40	40

Glucose Concentration (g/L)	>2.0	>2.0

Given these benefits, a cell culture comparison test between this new rocking design and a conventional, single axis rocking bioreactor was performed to evaluate the cell culture performance.

## Results

**Figure 1:**
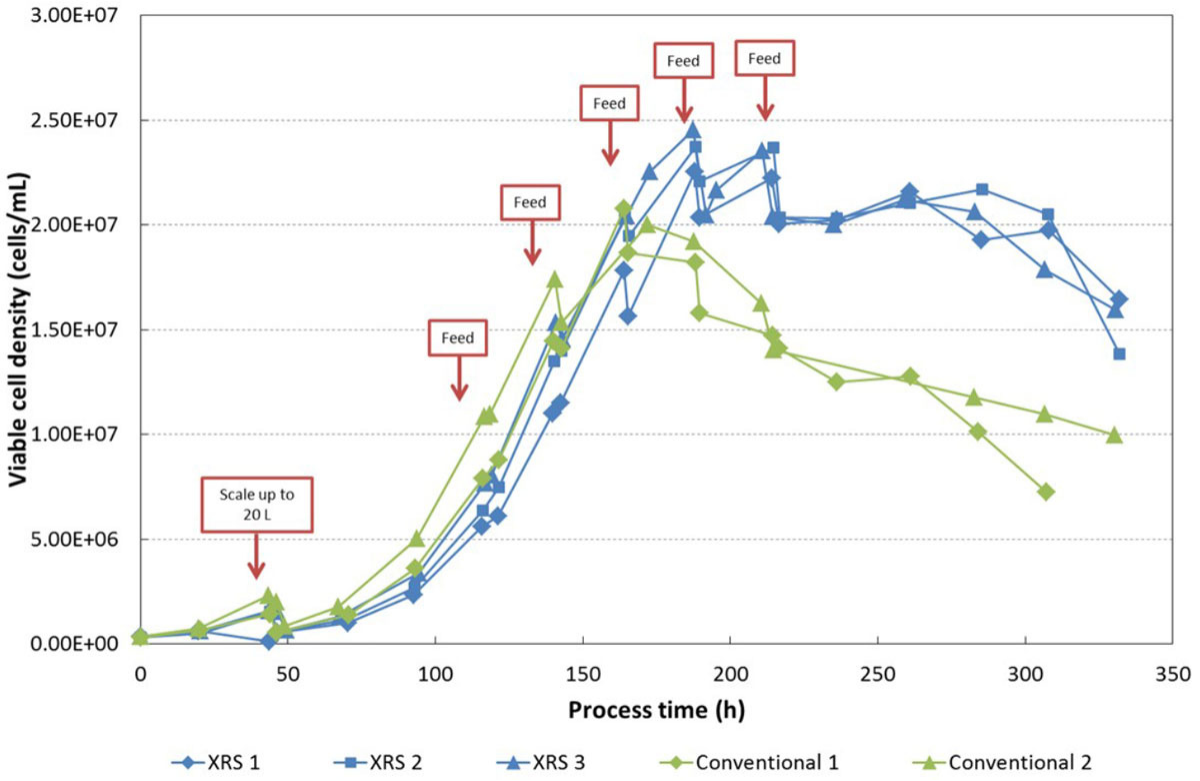
**Cell growth in the XRS 20 with bi-axial rocking and in conventional rocking bioreactors**.

Cultures from both the conventional rocking bioreactor and the Allegro XRS 20 system grew at a similar rate up until hour 165 at which point cells in the conventional device entered the stationary phase, data shown in figure 1. Cell growth continued in the XRS 20 bioreactor for a further24 hours. This resulted in an average maximum cell concentration of 2.36 × 107 viable cells/mL, an increase of 16% over the conventional rocker average.

Using a fed-batch process, the Pall XRS 20 System yielded a higher maximum viable cell density, for a longer duration. This was reflected in the end monoclonal antibody concentration from each reactor: on average there was a 67% increase in target antibody production in the Allegro XRS 20 System over the conventional bioreactor.

## Conclusions

We have demonstrated that the Allegro XRS 20 System is ideally suited to the cultivation of CHO cells, and compares favorably to the rocker system for this process. This is likely due to the unique design features built into this system. Therefore, the results confirm expectations, shown by improved cell titers and increased antibody production without compromising on product quality

